# Comparative Accuracy of Anal and Cervical Cytology in Screening for Moderate to Severe Dysplasia by Magnification Guided Punch Biopsy: A Meta-Analysis

**DOI:** 10.1371/journal.pone.0024946

**Published:** 2011-09-19

**Authors:** Wm. Christopher Mathews, Wollelaw Agmas, Edward Cachay

**Affiliations:** Department of Medicine, University of California San Diego, San Diego, California, United States of America; University of Ottawa, Canada

## Abstract

**Background:**

The accuracy of screening for anal cancer precursors relative to screening for cervical cancer precursors has not been systematically examined. The aim of the current meta-analysis was to compare the relative accuracy of anal cytology to cervical cytology in discriminating between histopathologic high grade and lesser grades of dysplasia when the reference standard biopsy is obtained using colposcope magnification.

**Methods and Findings:**

The outcome metric of discrimination was the receiver operating characteristic (ROC) curve area. Random effects meta-analysis of eligible studies was performed with examination of sources of heterogeneity that included QUADAS criteria and selected covariates, in meta-regression models. Thirty three cervical and eleven anal screening studies were found to be eligible. The primary meta-analytic comparison suggested that anal cytologic screening is somewhat less discriminating than cervical cytologic screening (ROC area [95% confidence interval (C.I.)]: 0.834 [0.809–0.859] vs. 0.700 [0.664–0.735] for cervical and anal screening, respectively). This finding was robust when examined in meta-regression models of covariates differentially distributed by screening setting (anal, cervical).

**Conclusions:**

Anal cytologic screening is somewhat less discriminating than cervical cytologic screening. Heterogeneity of estimates within each screening setting suggests that other factors influence estimates of screening accuracy. Among these are sampling and interpretation errors involving both cytology and biopsy as well as operator skill and experience.

## Introduction

The accuracy of screening procedures for anal cancer and its precursors relative to comparable procedures used in screening for cervical cancer and its precursors has not been systematically defined. The issue is of importance because invasive anal cancer rates are increasing among HIV-infected persons[1], [2] and because screening programs modeled on procedures used in cervical cancer screening are being increasingly implemented among persons at increased risk for anal cancer[3]. The primary objective of this study was to meta-analytically compare a summary operating characteristic of the performance of cervical and anal cytology testing in the detection of cervical and anal cancer and their precursors, when the reference standard biopsy is obtained at colposcopy or high resolution anoscopy (HRA), respectively.

## Methods

### Data Sources and Searches

#### Cervical

Data sources included: (1) MEDLINE from 2000 through 2010; (2) review of two previously published meta-analyses of cervical cytology accuracy.[4], [5] The MEDLINE search strategy included the following search terms in any field: *cervical cytology* AND *sensitivity* AND *specificity* AND *cervical intraepithelial neoplasia* AND *year of publication 2000-2010*. Searches were limited to English-language publications. Unpublished studies were ineligible for inclusion. Authors were not contacted to provide data in the eligible format if the published manuscript had incomplete data for analysis.

#### Anal

Data sources included: (1) MEDLINE from 1990 through 2010; (2) review of published systematic reviews [6]–[9]. Because the search algorithm comparable to that used for cervical publications yielded only 33 potentially relevant publications, the search strategy for anal publications was broadened. The MEDLINE search included the following search strategy in any field: (*anal cancer* OR *anal dysplasia* OR *anal cytology* OR *anal intraepithelial neoplasia*) AND *screening* AND ((*sensitivity* AND *specificity*) OR *accuracy*). Unpublished and non-English language publications were ineligible for inclusion. When a publication appeared to be eligible for inclusion but the data presented in the manuscript was incomplete, authors were contacted to request data in the required format (see below).

### Study Selection

In establishing study inclusion and exclusion criteria we used the following definitions of index and reference tests. The *index test* was cytologic sampling of cervicovaginal or anal canal tissues using cytology swabs or brushes and processing of the samples using either traditional slide fixation or liquid cytologic media. The *reference standard* was defined as colposcope magnified and directed punch biopsy of the uterine cervix or anal canal, respectively. Operator visual impression *without a biopsy* could not be included as part of the definition of the reference standard result. Inclusion criteria included published reports: (1) of primary screening or follow up evaluation for previous cytologic abnormalities; (2) use of the Bethesda 1991 or 2001 Classification System (or equivalent); (3) reference standard diagnosis by cervical or anal punch biopsy obtained using colposcope magnification; the addition of endocervical curettage sampling was allowed for colposcopy studies; (4) average time interval between cytology and punch biopsy ≤3 months; (5) availability of extractable data in the format below (Table 1), where “cases” are defined as those with histopathologic evidence by punch biopsy (cervical or anal) of cervical or anal intraepithelial neoplasia 2 (CIN 2 or AIN 2) or greater and cytology diagnostic categories include negative (“no atypical or malignant cells”), atypical squamous cells of uncertain significance (ASCUS), atypical squamous cells can't rule out high grade (ASC-H), low grade squamous intraepithelial lesion (LSIL), and high grade intraepithelial lesion (HSIL):

**Table 1:** Data format extracted for each included study in the present meta-analysis.

	Cytology			
Biopsy	Negative	ASCUS	LSIL	≥HSIL or ASC-H
≥CIN 2 (AIN 2)				
<CIN 2 (AIN 2)				

Exclusion criteria included: (1) reference standard established only by visual inspection at colposcopy or anoscopy without biopsy; (2) patients with normal colposcope magnified visual impression but no biopsy were classified as normal histology; (3) cervical cytology study sample explicitly included patients previously treated by conization or LLETZ (because of unavailability of comparable study populations for anal cytology studies).

### Data Extraction

In abstracting cytology data from the included publications, the following conventions were followed: (1) inflammatory changes were categorized with the “negative” category; (2) AGUS was categorized with ASCUS; (3) HPV changes or koilocytes were classified under LSIL; (4) the category “≥HSIL” included ASC-H, HSIL, CIS, and invasive carcinoma.

The *main outcome measure* was the receiver operating characteristic (ROC) curve area estimated from the extracted 2 by 4 data tables, wherein the cytology diagnostic categories are treated as ordinal measures and the reference standard is binary. An ROC metric to summarize the ability of cervical (anal) cytology to discriminate between ≥CIN 2 (AIN 2) and < CIN 2 (AIN 2) histology has been previously used [10]–[12] and has the advantage of not being cut point dependent.

Each identified publication was reviewed by a single reviewer (WCM) and classified as ineligible or potentially eligible based on review of the title and abstract. When ineligibility was in doubt, full reports were reviewed for a final determination. Full reports of potentially eligible publications were then independently reviewed by two investigators. Final eligibility determination was based on consensus of the two reviewers. Decisions regarding final eligibility for inclusion were made without knowledge of the cytology ROC area, which is the primary outcome metric of the meta-analysis.

### Validity Assessment: Study Quality and Covariate Rating

Eligible publications were reviewed using the QUADAS tool [13]–[15]. Further specification of quality and covariate review criteria was operationalized using the following additional questions:

1. Were patients undergoing the reference standard procedure (colposcopy or HRA directed punch biopsy) selected on the basis of prior screening cytology results? (Yes/No/Unclear)[verification bias]
2. What was the time interval between the test cytology and the reference standard procedure? (same day/ not same day but within 3 months/Unclear)[disease progression bias]
3. Was the reference standard result based only on punch biopsy interpretation? (punch biopsy only/composite of punch biopsy and colposcopy-HRA visual impression/mixture of punch biopsy and other histology/Unclear)[reference standard definition]
4. What cytology method was used? (Conventional/Thin Prep/Other liquid cytology/Unclear)
5. Were histologic and/or cytologic results reviewed for final classification by central adjudication or independent readers? (Yes/No/Unclear)
6. Did the study sample explicitly include HIV infected patients? (Yes/No/Unclear)
7. What was the study design? (Clinical Cohort/Case Control/Convenience sample of matched cytology and histology results/Clinical Trial/Other/Unclear)
8. What cytology classification system was used? (Bethesda 1991/Bethesda 2001/Other comparable/Other not comparable/Unclear)

The study quality review was separately scored by two co-authors (WCM and WA). Discordant ratings were resolved by consensus. Initial agreement (prior to consensus review) among reviewers was summarized using the prevalence and biased adjusted kappa statistic (PABAK)[16] implemented in WinPEPI version 11.4.[17]

### Data Synthesis and Analysis

For each eligible and included study, the diagnostic ROC area was estimated from extracted 2 by 4 raw frequency tables using the *roctab* procedure in Stata version 11.2 (StataCorp, College Station, TX). Estimated ROC areas with their standard errors (s.e.) were then pooled using the random effects model of DerSimonian and Laird[18] as implanted in the Stata *metan* procedure. The primary analysis included all eligible studies. A secondary planned analysis was performed conditioning on two factors that may influence comparability of the two screening contexts: (1) the time interval between most recent cytology and biopsy; and (2) the operative method for obtaining the histology reference standard. For this secondary analysis, included studies were limited to those reporting both same day cytology and histology reference standard based exclusively on colposcope directed punch biopsy.

Heterogeneity of effects was evaluated using the I^2^-statistic[19] and further explored with funnel plots graphing ROC area against s.e.(ROC) to detect asymmetry suggesting bias. The Egger test was performed as a quantitative test of skewness in the funnel plot.[20] Finally, we performed random effects meta-regression as implemented using the Stata *metareg* procedure including as covariates those QUADAS and covariate measures that were differentially distributed across screening setting (p<0.20) in contingency table analysis. Covariates found to be associated with the ROC outcome (p<0.05) in metagression models jointly adjusted for screening setting (cervical, anal) were used as stratification factors to estimate the effect of screening setting (cervical, anal) within levels of the same covariates.

## Results

### Trial Flow: Cervical Cytology-Biopsy Studies

**Figure 1:**
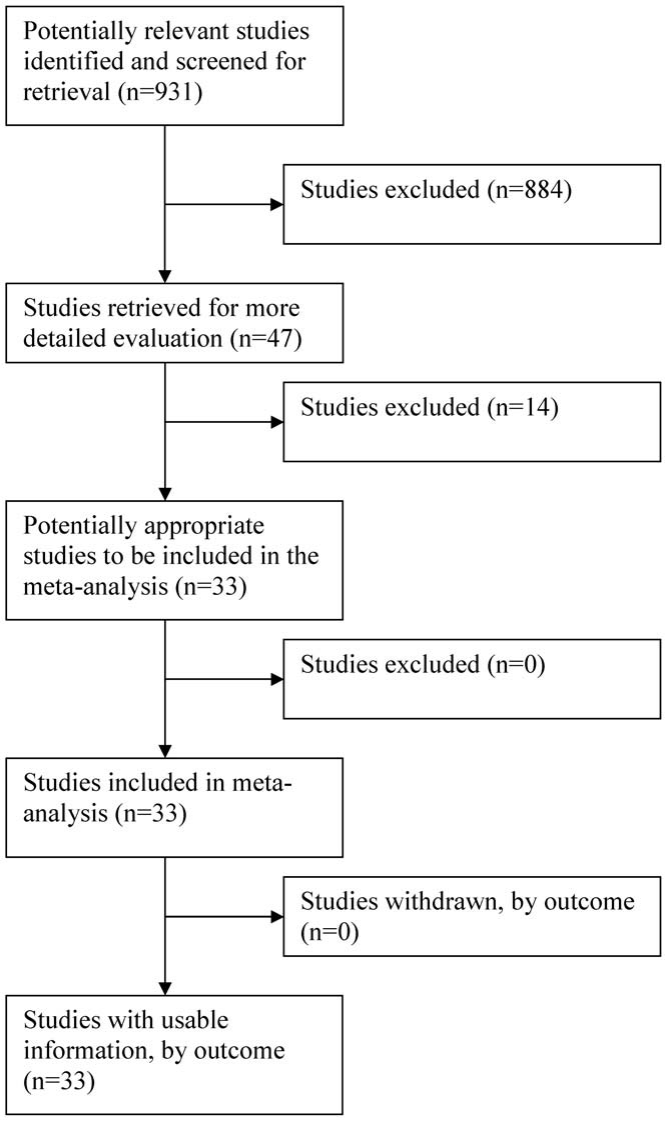
Flow of Included Studies: Cervical Screening.

From the MEDLINE search algorithm for cervical studies, 931 unduplicated publications were initially identified. Primary reasons for exclusion of 884 publications on initial screen included: (1) lack of relevance to the research question; (2) incomplete data for analysis evident by review of abstract; and (3) use of cytology system not comparable to Bethesda system. The remaining 47 publications were judged to be potentially eligible for study inclusion, and their manuscripts were independently reviewed for final determination of eligibility by two investigators (WCM and WA). Of these, 14 were excluded for the following reasons (more than one may apply to each study): (1) non-biopsied patients were classified as having normal histology if colposcopic appearance was normal (n = 4); (2) cytology-biopsy interval either not stated or exceeded average of 3 months (n = 8); (3) reported data aggregated across cytology categories (n = 2); (4) cytology classification not comparable to Bethesda system (n = 1); and (5) substantial missing data (31/52) with small sample size (n = 1). The thirty three remaining studies met eligibility criteria and were included (Figure 1).

### Trial Flow: Anal Cytology-Biopsy Studies

**Figure 2:**
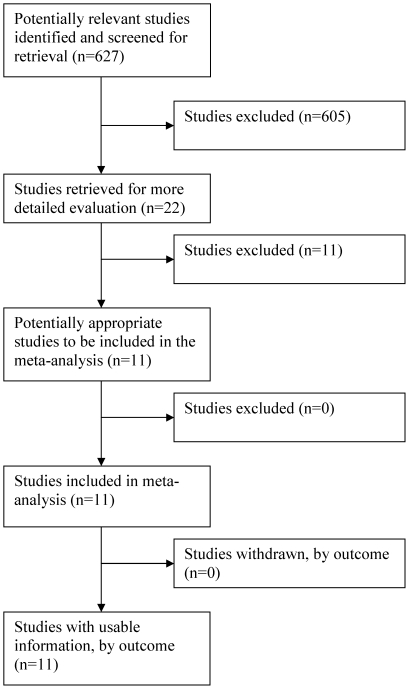
Flow of Included Studies: Anal Screening.

From the MEDLINE search strategy for anal publications, 627 unduplicated publications were initially identified. Of these, 605 were excluded on preliminary screen. Primary reasons for exclusion included: (1) lack of relevance to the research question; (2) lack of primary data (e.g. review articles); (3) incomplete data collected as evident from review of the abstract (e.g. investigators did not use colposcopic magnification or range of studied cytology results restricted). The remaining 22 studies were judged to be potentially eligible. Of the 22, 11 were excluded after detailed review and attempts to contact authors for clarification or data. Nine authors were emailed with requests to provide data in the required format. Of the 9, 4 responded and 1 was able to provide eligible data. Reasons for final exclusion of the 11 studies (more than one category may apply to each study) were: (1) incomplete data for analysis (n = 8); (2) cytology-biopsy interval either not stated or exceeded average of 3 months (n = 5); (3) HRA not performed in all reported cases (n = 2); and (4) cytology classification not comparable to Bethesda system (n = 1). The final number of included eligible studies was eleven (Figure 2).

### Study Characteristics: Study Quality and Covariate Review

**Figure 3:**
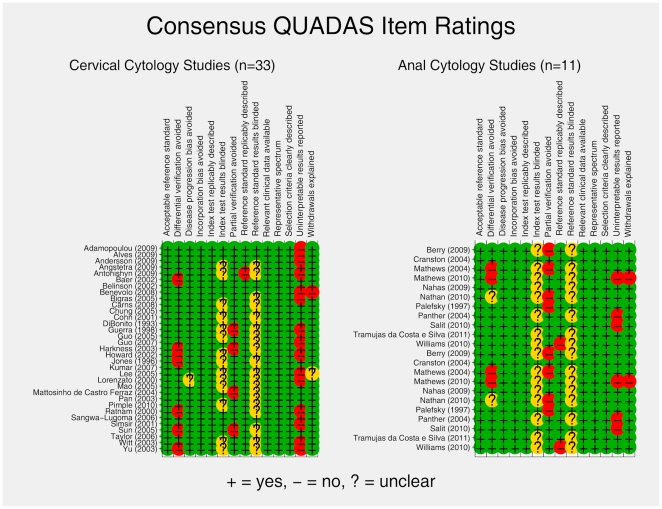
Consensus QUADAS ratings by study category (Cervical or Anal).

PABAK agreement (median [range]) between the two primary study reviewers on QUADAS item scoring was 0.72 [0.44 – 0.94] and 0.73 [0 – 0.82], for cervical and anal cytology studies, respectively. PABAK was not estimable for those items for which reviewers uniformly chose a single rating option. Consensus QUADAS ratings by study category (cervical or anal) are presented in Figure 3. Reviewers judged that study participants were selected based on prior cytology screening results in 58% and 27% (p = 0.192) of cervical and anal cytology studies, respectively. There was no difference in the distribution of cytology-biopsy time intervals comparing cervical to anal studies, with 71% and 82% (p = 0.774) of studies reporting same day cytology and biopsy measures, respectively. With regard to the histological reference standard, 91% of anal studies reported exclusive use of HRA directed punch biopsy whereas 65% of the cervical studies reported exclusive use of colposcopically directed punch biopsy (p = 0.344). The most common additional histological reference standard component included in the cervical studies was endocervical curettage. There was no difference in reported cytological method between the two study types with 65% and 64% (p = 0.364) of cervical and anal studies reporting use of conventional cytology, respectively. Only 3% of cervical studies reported on HIV-infected participants, whereas 100% of anal studies included (not necessarily exclusively) HIV infected persons. By study design, 80% of the cervical studies were cohort designs in comparison 91% of anal studies; 12% and 9% of cervical and anal studies, respectively, were cross sectional studies involving matching of available cytology to biopsy results. The Bethesda 1991 cytology system was reported in 71% and 36% (p = 0.12) of cervical and anal studies, respectively. This difference reflects the more recent publication of HRA studies.

### Quantitative Data Synthesis

**Figure 4:**
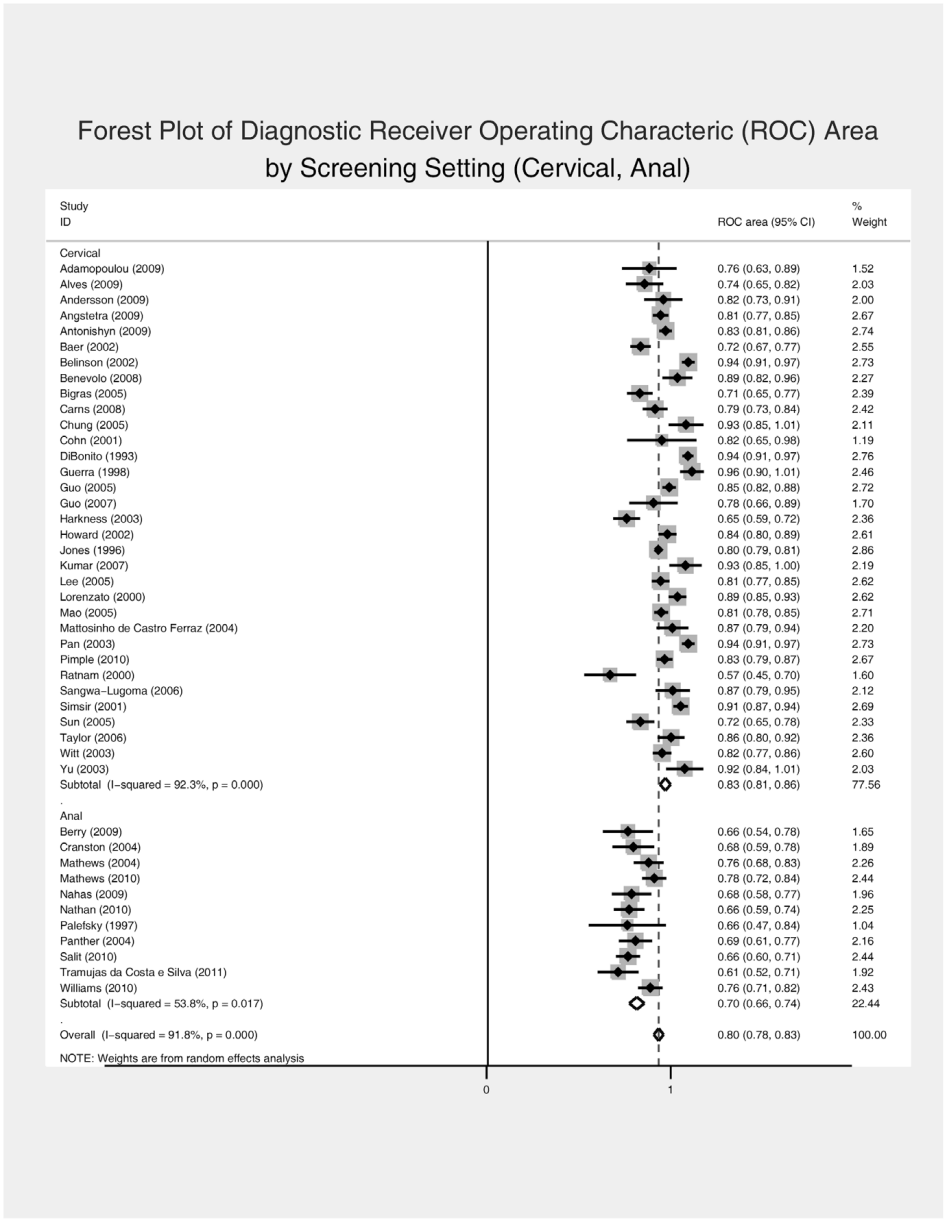
Forest Plot of Diagnostic Receiver Operating Characteric (ROC) Area, by Screening Setting (Cervical, Anal).

**Figure 5:**
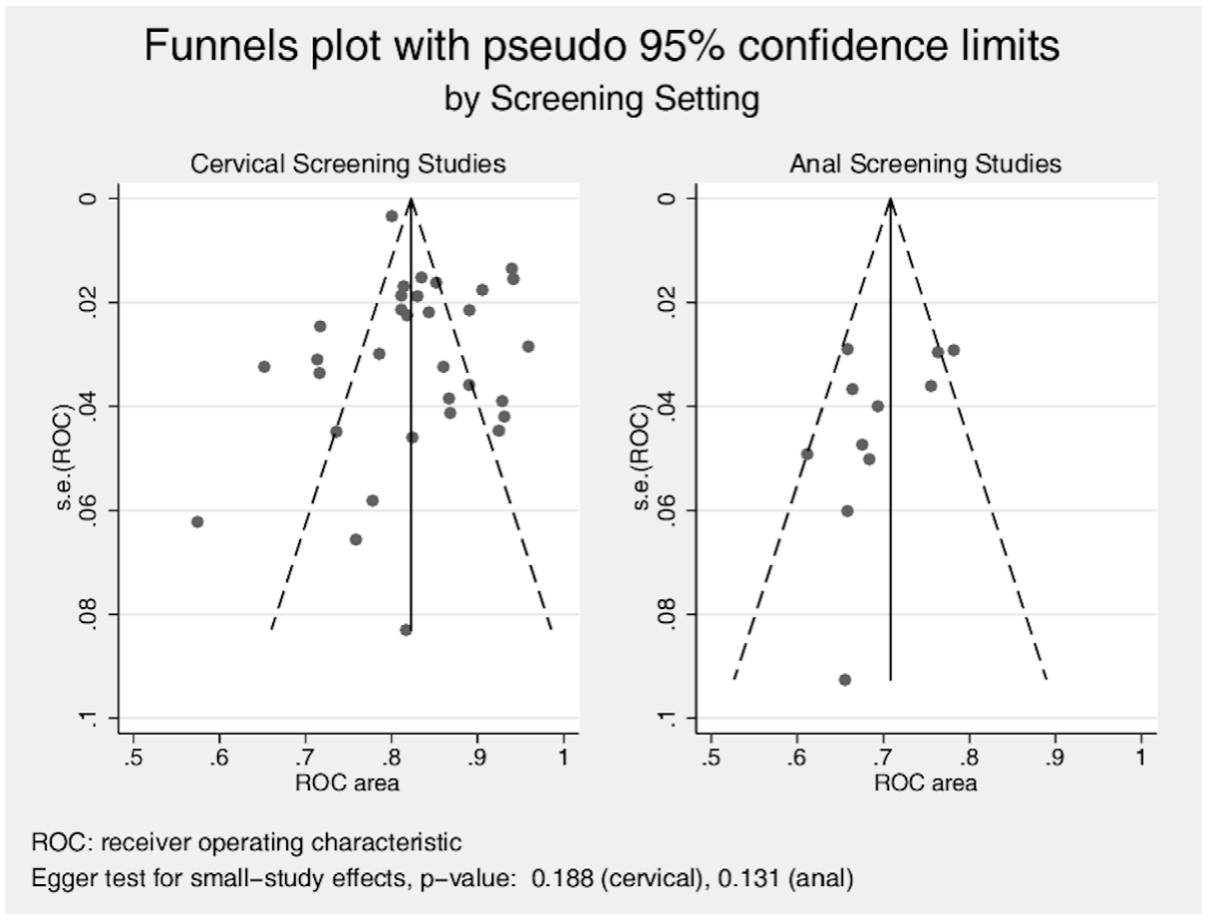
Funnel plots with pseudo 95% confidence limits, by Screening Setting.

Table S1 presents the data extraction results and summary metric (cytology-biopsy ROC area) organized by study type (cervical and anal). The primary analysis (Figure 4) comparing the ability of cervical and anal cytology to discriminate between high grade and non-high grade histology by colposcope directed biopsy suggested superiority for cervical screening (ROC area [95% C.I.]: 0.834 [0.809 – 0.859] vs. 0.700 [0.664 – 0.735] for cervical and anal screening, respectively). While heterogeneity of effect was evident among studies for both screening contexts, it was greater among cervical screening studies (I^2^ statistic 92.3%, p<0.0001 for cervical studies and 53.8%, p = 0.017 for anal studies). This difference in heterogeneity across screening context is also evident in the funnel plots (Figure 5), which demonstrate that relatively more of the cervical screening studies fall outside the pseudo 95% confidence intervals than is observed for the anal screening studies. The relative symmetry of both funnel plots is supported by the non-significant Egger test for both.

When the analysis was restricted to those studies reporting both concurrent (same day) cytology and biopsy and also reporting histology reference standard obtained exclusively using punch biopsy (excluding endocervical curettage), the difference between screening contexts was greater than that estimated for the unrestricted primary analysis (ROC area [95% C.I.]: 0.871 [0.844 – 0.899] vs. 0.690 [0.649 – 0.732] for cervical and anal screening, respectively).

The distributions of the following QUADAS items and covariates were differential (p<0.20) in contingency table analyses of study characteristics and screening setting: (1) QUADAS 5 (partial verification bias)[p = 0.071], (2) QUADAS 10 (index test blinded)[p = 0.082], (3) patients referred for biopsy on the basis of prior screening cytology [p = 0.087],(4) HIV-infected patients included in study [p<0.0001], and (5) the cytology classification system used [p = 0.092]. Each of these five characteristics was entered as a categorical indicator in separate random effects meta-regressions that included also screening setting (cervical, anal) to identify those that were explanatory of heterogeneity in estimated ROC outcome. Of these five study characteristics, only the item dealing with referral for colposcope directed biopsy conditional upon screening cytology results was significantly associated with estimates of ROC heterogeneity in random effects meta-regression models (Table 2, Model 3). It is also evident in Table 2 that, with the exception of Model 4 (HIV+ patients included), the estimated effect of screening setting remained statistically significant and in all models consistent with the overall effect screening setting favoring cervical screening over anal screening. Regarding Model 4, it should be noted that only 1 of 32 cervical screening studies explicitly included HIV infected patients whereas all 11 of the anal screening studies included HIV infected patients. So the effect of screening setting was strongly confounded with the HIV covariate.

**Table 2:** Meta-Regression Estimates of Joint Effects of Screening Setting and Selected Covariates (n = 44 studies).

Model	Covariate	b[covariate]	s.e.[covariate	p[covariate]	b[screening setting_3_]	s.e.[screening setting]	p[screening setting]
**1**	QUADAS 5_1_ [reference = yes]	--					
	QUADAS 5 = no	-0.028	0.034	0.419	-0.132	0.03	<0.0001
**2**	QUADAS 10_2_ [reference: yes]	--					
	QUADAS 10 = unclear	0.2	0.025	0.445	-0.143	0.03	<0.0001
**3**	Conditional referral for biopsy [reference: yes]			0.045			
	no	0.061	0.026	0.023	-0.153	0.028	<0.0001
	unclear	0.061	0.035	0.085			
**4**	HIV+ patients included [reference: yes]	--					
	no	0.016	0.112	0.885	-0.121	0.114	0.296
**5**	Cytology Classification System [reference: Bethesda 1991]	--		0.836			
	Bethesda 2001	-0.01	0.029	0.726	-0.131	0.032	<0.0001
	Other comparable	-0.032	0.059	0.588			

1. QUADAS 5. Partial verification avoided.

2. QUADAS 10. Index test results blinded.

3. Screening setting: anal. Reference: cervical.

Because Model 3 (referral for biopsy conditional upon screening cytology results) suggested that conditional referral was associated with at least part of the observed effect heterogeneity in the primary meta analytic result, we re-estimated the screening setting effects for each of the three rating options for conditional referral (yes, no, unclear). The estimated pooled ROC effects for screening setting restricting eligibility to those studies for which the conditional referral item was rated “yes” were: 0.797 (95% CI: 0.771–0.822) for cervical screening (n = 19 studies) and 0.749 (0.699–0.798) for anal screening (n = 3 studies), with associated I^2^ heterogeneity estimates of 86.3% and 37.3%, respectively. The corresponding pooled ROC effect estimates restricting eligibility to studies for which the conditional referral item was rates “no” were: 0.898 (0.865–0.931) for cervical screening (n = 10 studies) and 0.657 (0.621–0.694) for anal screening (n = 6 studies), with associated I^2^ estimates of 74.9% and 0%, respectively. Finally for the conditional referral rating of “unclear” for which there were only 4 eligible cervical and 2 eligible anal studies, the corresponding pooled ROC estimates were 0.868 (0.785–0.951) and 0.716 (0.618–0.813) with I^2^ estimates of 91.8% and 77.5%, respectively. Thus, in these exploratory analyses based on meta-regression results, non-overlapping ROC confidence intervals were observed only for the conditional referral category of “no” while the subgroup study sample size was considerably reduced for each restricted comparison.

## Discussion

To our knowledge, this study is the first attempt to systematically compare the relative accuracy of anal and cervical screening for cancer precursors. In the primary meta-analysis of 33 cervical and 11 anal screening studies, we found that anal cytologic screening appeared to be somewhat less discriminating than cervical cytologic screening for detecting high grade histopathologic lesions (≥CIN 2 or AIN 2): (1) when the index of discrimination is defined as area under the receiver operating characteristic (ROC) curve; (2) when the reference standard biopsy is obtained using colposcope magnification; and (3) when the interval between cytology and biopsy is less than or equal to 3 months. This conclusion was robust when restricting study eligibility by requiring that cytology and histology be ascertained on the same day using only punch biopsy to obtain the reference standard diagnosis. Although there was considerable heterogeneity among both cervical and anal screening studies (more so among cervical studies), we found that the primary result was confirmed in meta-regression models controlling for those study quality indicators and covariates that were differentially associated with screening setting (cervical, anal).

A number of factors may account for or contribute to the primary meta-analytic finding. First is the possibility that the currently used screening procedures for anal cancer and its precursors are intrinsically less accurate than the comparable procedures for cervical screening. Cytology is obtained blindly in anal screening and under direct visualization in cervical screening. In addition, obtaining the reference standard biopsy is more challenging in the anal canal because of the collapsing nature of the organ such that lesions may be obscured by tissue folds not adequately retracted [21].

However before concluding intrinsic inferiority of anal screening, sources of bias in the conduct of the meta-analysis must be examined. First, our study selection procedures differed by screening setting as discussed in the Methods section. We used expanded MEDLINE search terms to initially identify potentially eligible anal screening studies because of the low yield when applying the terms used to identify cervical screening studies. Second, we contacted authors for clarifying information and for formatted data for the potentially eligible anal studies but not for the cervical studies. Third, the response rate from contacted authors of anal screening studies was low. Fourth, we excluded studies (both cervical and anal) that examined other metrics of cytology screening performance (sensitivity, specificity, predictive value) when data inadequate to estimate our ROC metric were either not published or not made available upon data request (in the case of anal studies). We do not know whether these procedural decisions resulted in biased study selection and estimation of effects. However we required that decisions regarding study selection were made prior to estimating study ROC outcomes. Furthermore, examination of the funnel plot symmetry and non-significant Egger tests provide some support for a conclusion that selection of publications was not seriously biased.

The sample size (n = 21,616) of one cervical screening study, Jones (1996), far exceeded the sample sizes of all other included studies and would have dominated the analysis using a fixed effect weighting procedure. The median [range] sample sizes for the anal and cervical studies were 169 [75, 401] and 448 [54, 21616]. We chose random effects meta-analysis in order to allow estimation of the true effect to vary between studies and to give relatively greater weight to smaller studies.[22] The primary meta-analytic result was concordant for both random and fixed effect models; the pooled *fixed effect* ROC estimates (95% CI) for anal and cervical screening studies were 0.708 (0.685–0.731) and 0.823 (0.818–0.828), respectively.

Although a few of the included studies addressed the issue of *verification bias*, the raw data abstracted from all included studies was unadjusted for verification bias. The study raters judged that 58% of included cervical screening studies and 27% of anal studies were subject to potential verification bias in that referral for colposcopy or HRA was conditional upon screening cytology results. Although conditional referral was identified in meta-regression analysis as significantly associated with ROC heterogeneity (Table 2, Model 3), controlling for it did not alter the primary meta-analytic result.

If one accepts our meta-analytic ROC estimates as valid, what conclusions can be drawn regarding screening for anal cancer precursors? First, we believe that the finding of somewhat less discriminatory ability for anal cytology than for cervical cytology does not diminish the rationale for anal cancer screening. The rationale has been argued cogently with an accumulating evidence base[7], [23] on the basis of accepted criteria for public health screening[24], albeit with considerable uncertainty regarding the efficacy of treatments for anal cancer precursors identified through screening and also regarding screening logistics (who to screen, how to screen, how often to screen).[8] Second, experience with performing anal cytology and HRA is much more limited and recent than comparable experience screening for cervical cancer and its precursors. It is known that operator experience makes a difference [25] and HRA investigators and practitioners are still learning to optimize techniques.[26], [27] Heterogeneity of ROC estimates may in part reflect operator experience and skill such that the ceiling for accuracy of combined screening with cytology, HRA, and biopsy components may not yet have been achieved. Third, studies of accuracy of both cervical and anal screening procedures should take into account the fact that the reference standard of punch biopsy is itself an imperfect reference standard subject to sampling and interpretation error[28] just as is the case for cytological screening.[21] Fourth, a recent cost-effectiveness analysis of screening for anal cancer precursors in HIV-infected men having sex with men concluded that direct use of HRA was the most cost-effective strategy for detecting AIN 2/3 that high risk population.[29] It is our view, however, that because that conclusion assumed that HRA directed biopsy was itself a perfect reference standard and did not take into account the potential value of repeated cytological examination combined with digital rectal examination, our finding of somewhat less discriminating performance of anal cytology in comparison with cervical cytology does not of itself support a recommendation to eliminate prior cytology screening as a component of screening for anal cancer precursors in high risk populations. In conclusion, we believe that our results better define the relative accuracy of screening for anal cancer precursors and contribute to ongoing policy discussions regarding formulation of guidelines regarding anal cancer screening in populations at risk.

## Supporting Information

Table S1 Extracted Study Data and Outcome Metrics, by Study Type Cytology-Biopsy Joint Cell Frequencies.
(DOCX)
