# Supplementary material for: Comparative Accuracy of Anal and Cervical Cytology in Screening for Moderate to Severe Dysplasia by Magnification Guided Punch Biopsy: A Meta-Analysis
Source: PLoS One. 2011 Sep 19;6(9):e24946. doi: 10.1371/journal.pone.0024946 (PMC3176293; doi:10.1371/journal.pone.0024946)
Supplement: Table S1 — Extracted Study Data and Outcome Metrics, by Study Type Cytology-Biopsy Joint Cell Frequencies. (DOCX) [file pone.0024946.s001.docx]

**Table S1: Extracted Study Data and Outcome Metrics, by Study Type Cytology-Biopsy Joint Cell Frequencies_1_**

|  |  |  |  |  |  | | | | | | | | | | | | | | | |
| --- | --- | --- | --- | --- | --- | --- | --- | --- | --- | --- | --- | --- | --- | --- | --- | --- | --- | --- | --- | --- |
| **Study Type** | **Study ID** | **n** | **ROC area_2_** | **s.e (ROC)** | **Negative,**  **< HSIL** | **Negative,**  **≥ HSIL** | **ASCUS,**  **< HSIL** | | | **ASCUS,**  **≥ HSIL** | **LSIL,**  **< HSIL** | | | **LSIL,**  **≥ HSIL** | | **HSIL or ASC/H, <HSIL** | | | **HSIL or ASC/H, ≥ HSIL** |  |
| **Cervical** |  |  |  |  |  |  |  | | |  |  | | |  | |  | | |  |  |
|  | Adamopoulou (2009)[[30](#_ENREF_30)] | 62 | 0.7586 | 0.0656 | 0 | 0 | 2 | | | 4 | 24 | | | 1 | | 7 | | | 24 |  |
|  | Alves (2009)[[31](#_ENREF_31)] | 115 | 0.7359 | 0.0449 | 20 | 6 | 15 | | | 4 | 12 | | | 3 | | 17 | | | 38 |  |
|  | Andersson (2009)[[32](#_ENREF_32)] | 78 | 0.8243 | 0.046 | 8 | 3 | 1 | | | 1 | 19 | | | 8 | | 3 | | | 35 |  |
|  | Angstetra (2009)[[33](#_ENREF_33)] | 485 | 0.8115 | 0.0187 | 121 | 8 | 26 | | | 7 | 98 | | | 10 | | 76 | | | 139 |  |
|  | Antonishyn (2009)[[34](#_ENREF_34)] | 887 | 0.8349 | 0.0152 | 361 | 41 | 90 | | | 20 | 130 | | | 60 | | 22 | | | 163 |  |
|  | Baer (2002)[[35](#_ENREF_35)] | 1075 | 0.7171 | 0.0246 | 498 | 41 | 262 | | | 27 | 115 | | | 22 | | 49 | | | 61 |  |
|  | Belinson (2002)[[36](#_ENREF_36)] | 1997 | 0.9415 | 0.0155 | 1486 | 5 | 302 | | | 6 | 81 | | | 9 | | 42 | | | 66 |  |
|  | Benevolo (2008)[[37](#_ENREF_37)] | 129 | 0.8904 | 0.0359 | 41 | 1 | 18 | | | 5 | 34 | | | 3 | | 1 | | | 26 |  |
|  | Bigras (2005)[[38](#_ENREF_38)] | 1533 | 0.7137 | 0.031 | 1135 | 34 | 158 | | | 13 | 149 | | | 10 | | 9 | | | 25 |  |
|  | Carns (2008)[[39](#_ENREF_39)] | 597 | 0.7858 | 0.0299 | 124 | 0 | 193 | | | 18 | 198 | | | 17 | | 14 | | | 33 |  |
|  | Chung (2005)[[40](#_ENREF_40)] | 54 | 0.9308 | 0.042 | 25 | 1 | 1 | | | 0 | 10 | | | 2 | | 1 | | | 14 |  |
|  | Cohn (2001)[[11](#_ENREF_11)] | 101 | 0.817 | 0.083 | 63 | 2 | 13 | | | 1 | 14 | | | 3 | | 1 | | | 4 |  |
|  | DiBonito (1993)[[41](#_ENREF_41)] | 916 | 0.9396 | 0.0135 | 677 | 4 | 48 | | | 8 | 81 | | | 15 | | 20 | | | 63 |  |
|  | Guerra (1998)[[42](#_ENREF_42)] | 72 | 0.9588 | 0.0285 | 7 | 0 | 2 | | | 0 | 9 | | | 3 | | 1 | | | 50 |  |
|  | Guo (2005)[[43](#_ENREF_43)] | 782 | 0.8521 | 0.0162 | 160 | 8 | 109 | | | 16 | 269 | | | 42 | | 26 | | | 152 |  |
|  | Guo (2007)[[44](#_ENREF_44)] | 92 | 0.778 | 0.0581 | 3 | 0 | 46 | | | 5 | 17 | | | 6 | | 5 | | | 10 |  |
|  | Harkness (2003)[[45](#_ENREF_45)] | 282 | 0.652 | 0.0324 | 49 | 19 | 42 | | | 25 | 52 | | | 29 | | 20 | | | 46 |  |
|  | Howard (2002)[[46](#_ENREF_46)] | 384 | 0.8435 | 0.0219 | 66 | 3 | 85 | | | 3 | 100 | | | 22 | | 35 | | | 70 |  |
|  | Jones (1996)[[47](#_ENREF_47)] | 21616 | 0.8004 | 0.0034 | 3900 | 311 | 3689 | | | 616 | 6914 | | | 1571 | | 979 | | | 3636 |  |
|  | Kumar (2007)[[48](#_ENREF_48)] | 124 | 0.9284 | 0.039 | 91 | 1 | 7 | | | 1 | 5 | | | 0 | | 6 | | | 13 |  |
|  | Lee (2005)[[49](#_ENREF_49)] | 400 | 0.8115 | 0.0214 | 77 | 26 | 41 | | | 21 | 65 | | | 26 | | 8 | | | 136 |  |
|  | Lorenzato (2000)[[50](#_ENREF_50)] | 448 | 0.8907 | 0.0215 | 284 | 11 | 32 | | | 6 | 31 | | | 11 | | 13 | | | 60 |  |
|  | Mao (2005)[[51](#_ENREF_51)] | 1266 | 0.8142 | 0.0169 | 729 | 40 | 147 | | | 29 | 128 | | | 41 | | 48 | | | 104 |  |
|  | Mattosinho de Castro Ferraz (2004)[[52](#_ENREF_52)] | 226 | 0.8669 | 0.0385 | 137 | 4 | 2 | | | 2 | 45 | | | 3 | | 10 | | | 23 |  |
|  | Pan (2003)[[53](#_ENREF_53)] | 1993 | 0.9412 | 0.0155 | 1475 | 5 | 308 | | | 6 | 82 | | | 9 | | 42 | | | 66 |  |
|  | Pimple (2010)[[54](#_ENREF_54)] | 1931 | 0.8302 | 0.0188 | 1710 | 52 | 22 | | | 9 | 26 | | | 8 | | 11 | | | 93 |  |
|  | Ratnam (2000)[[55](#_ENREF_55)] | 128 | 0.5743 | 0.0622 | 50 | 14 | 25 | | | 4 | 18 | | | 5 | | 5 | | | 7 |  |
|  | Sangwa-Lugoma (2006)[[56](#_ENREF_56)] | 526 | 0.8684 | 0.0413 | 459 | 6 | 18 | | | 2 | 12 | | | 7 | | 9 | | | 13 |  |
|  | Simsir (2001)[[57](#_ENREF_57)] | 852 | 0.9058 | 0.0176 | 370 | 7 | 182 | | | 7 | 158 | | | 14 | | 23 | | | 91 |  |
|  | Sun (2005)[[58](#_ENREF_58)] | 876 | 0.7163 | 0.0336 | 73 | 10 | 549 | | | 23 | 161 | | | 36 | | 1 | | | 23 |  |
|  | Taylor (2006)[[59](#_ENREF_59)] | 2435 | 0.8605 | 0.0324 | 2033 | 9 | 224 | | | 7 | 74 | | | 6 | | 58 | | | 24 |  |
|  | Witt (2003)[[60](#_ENREF_60)] | 280 | 0.8183 | 0.0225 | 28 | 5 | 3 | | | 6 | 74 | | | 49 | | 6 | | | 109 |  |
|  | Yu (2003)[[61](#_ENREF_61)] | 265 | 0.9247 | 0.0447 | 234 | 2 | 12 | | | 4 | 3 | | | 0 | | 0 | | | 10 |  |
| **Anal** |  |  |  |  |  |  |  | | |  |  | | |  | |  | | |  |  |
|  | Berry (2009)[[62](#_ENREF_62)] | 75 | 0.6579 | 0.0601 | 22 | 11 | 4 | | | 7 | 8 | | | 8 | | 4 | | | 11 |  |
|  | Cranston (2004)[[63](#_ENREF_63)] | 101 | 0.6834 | 0.0502 | 15 | 18 | 1 | | | 2 | 14 | | | 22 | | 2 | | | 27 |  |
|  | Mathews (2004)[[64](#_ENREF_64)] | 154 | 0.7552 | 0.0361 | 7 | 0 | 17 | | | 7 | 33 | | | 31 | | 10 | | | 49 |  |
|  | Mathews (2010)[[21](#_ENREF_21)] | 371 | 0.7816 | 0.0292 | 35 | 2 | 86 | | | 9 | 129 | | | 18 | | 37 | | | 55 |  |
|  | Nahas (2009)[[65](#_ENREF_65)] | 311 | 0.6751 | 0.0474 | 150 | 7 | 20 | | | 4 | 102 | | | 15 | | 8 | | | 5 |  |
|  | Nathan (2010)[[66](#_ENREF_66)] | 273 | 0.6637 | 0.0367 | 76 | 13 | 59 | | | 14 | 58 | | | 31 | | 10 | | | 12 |  |
|  | Palefsky (1997)[[67](#_ENREF_67)] | 135 | 0.6551 | 0.0926 | 50 | 3 | 28 | | | 2 | 43 | | | 4 | | 2 | | | 3 |  |
|  | Panther (2004)[[68](#_ENREF_68)] | 153 | 0.6933 | 0.04 | 18 | 1 | 18 | | | 12 | 46 | | | 26 | | 8 | | | 24 |  |
|  | Salit (2010)[[69](#_ENREF_69)] | 401 | 0.6581 | 0.029 | 117 | 16 | 40 | | | 9 | 120 | | | 52 | | 26 | | | 21 |  |
|  | Tramujas da Costa e Silva (2011)[[70](#_ENREF_70)] | 169 | 0.6117 | 0.0492 | 109 | 19 | 7 | | | 0 | 18 | | | 11 | | 3 | | | 2 |  |
|  | Williams (2010)[[71](#_ENREF_71)] | 241 | 0.7632 | 0.0296 | 13 | 0 | 36 | | | 9 | 83 | | | 31 | | 16 | | | 53 |  |
|  |  |  |  |  |  | | |  |  | | |  |  | |  | |  |  | | |
| (1) ASCUS = atypical squamous cell of uncertain significance; LSIL = low grade squamous intra epithelial lesion; HSIL = high grade squamous intra epithelial lesion; ASC/H = atypical squamous cells, can't exclude high grade | | | | | | | | | | | | | | | | | | | | |
| (2) Receiver Operating Characteristic | | |  |  |  | | |  |  | | |  |  | |  | |  |  | | |
